# Adenocarcinoma arising from jejunal ectopic pancreas mimicking peritoneal metastasis from colon cancer: a case report and literature review

**DOI:** 10.1186/s40792-015-0118-1

**Published:** 2015-11-14

**Authors:** Yusuke Yamaoka, Tomohiro Yamaguchi, Yusuke Kinugasa, Akio Shiomi, Hiroyasu Kagawa, Yushi Yamakawa, Masakatsu Numata, Shinya Sugimoto, Kenichiro Imai, Kinichi Hotta, Keiko Sasaki

**Affiliations:** Division of Colon and Rectal Surgery, Shizuoka Cancer Center Hospital, 1007 Shimonagakubo, Nagaizumi-cho, Sunto-gun, Shizuoka 411-8777 Japan; Division of Endoscopy, Shizuoka Cancer Center Hospital, 1007 Shimonagakubo, Nagaizumi-cho, Sunto-gun, Shizuoka 411-8777 Japan; Division of Pathology, Shizuoka Cancer Center Hospital, 1007 Shimonagakubo, Nagaizumi-cho, Sunto-gun, Shizuoka 411-8777 Japan

**Keywords:** Ectopic, Heterotopic, Aberrant, Pancreas, Adenocarcinoma, Jejunum

## Abstract

Adenocarcinoma arising from jejunal ectopic pancreas is very rare. We report a case of a 69-year-old female with adenocarcinoma arising from jejunal ectopic pancreas after resection of advanced colon cancer. She underwent right hemicolectomy for advanced ascending colon cancer (ypT3N0M0, stage IIA) after chemotherapy. Two and half years after colectomy, her tumor markers were elevated, and computed tomography revealed a mass measuring 20 × 20 mm in the small intestine, having an abnormal uptake of ^18^F-fluorodeoxyglucose on ^18^F-fluorodeoxyglucose-positron emission tomography (^18^FDG-PET). Double-balloon enteroscopy revealed a submucosal tumor in the jejunum, and histopathology of biopsy specimens from that lesion showed ectopic pancreas without malignancy. Therefore, peritoneal metastasis from colon cancer concomitant with ectopic pancreas or adenocarcinoma arising from ectopic pancreas was considered as a differential diagnosis. She underwent laparoscopic jejunectomy. Pathological examination revealed a moderately differentiated adenocarcinoma arising from jejunal ectopic pancreas, not peritoneal metastasis from colon cancer. Even if histopathology of the biopsy specimen shows ectopic pancreas without malignancy, adenocarcinoma arising from ectopic pancreas should be considered when the tumor markers are elevated or the lesion has an abnormal uptake of ^18^FDG.

## Background

Ectopic pancreas is defined as pancreatic tissue lacking anatomical and vascular continuity with the normally located pancreas. Ectopic pancreas is usually an asymptomatic, incidental finding at laparotomy or autopsy, and its malignant transformation is extremely rare, especially in persons with jejunal ectopic pancreas [1–3]. Therefore, adenocarcinoma arising from ectopic pancreas is difficult to diagnose before resection. The appropriate extent of lymph node dissection, the effect of chemotherapy, and the prognosis remain unclear [4]. We herein report a case of adenocarcinoma arising from jejunal ectopic pancreas with peritoneal metastasis-like features that occurred after resection of advanced colorectal cancer.

## Case presentation

**Fig. 1:**
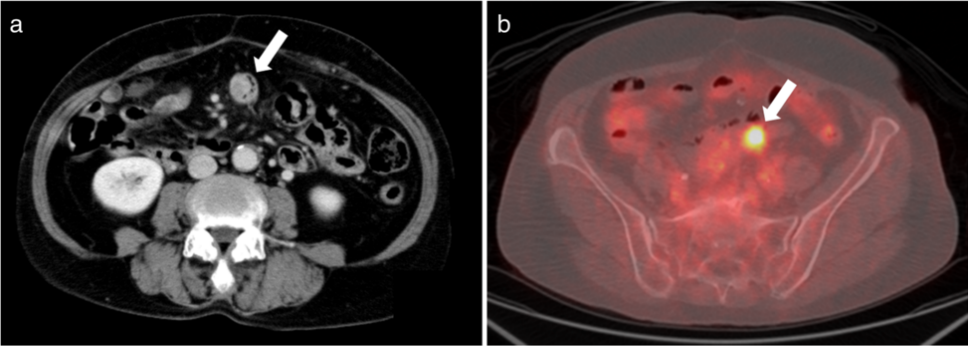
Computed tomography showed a mass measuring 20 × 20 mm in the small intestine (**a**) (*arrow*), and ^18^F-fluorodeoxyglucose (^18^FDG)-positron emission tomography/computed tomography showed an abnormal uptake of ^18^FDG in the lesion (**b**) (*arrow*)

**Fig. 2:**
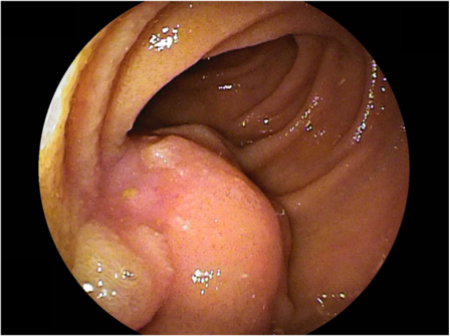
Double-balloon enteroscopy showed an approximately 2-cm submucosal tumor in the jejunum at 100 cm from the incisor teeth

A 65-year-old Japanese female underwent ileal-colonic bypass for unresectable ascending colon cancer with invasion to the duodenum and multiple pulmonary nodules, suspected to be pulmonary metastases. She was a current smoker and had no pertinent medical history. After she received 37 cycles of chemotherapy combining folinic acid, 5-fluorouracil, irinotecan, and bevacizumab (FOLFIRI+Bev), computed tomography (CT) showed a remarkable reduction in tumor size in the ascending colon. The sizes of the patient’s pulmonary nodules were not changed; hence, they were considered as benign lesions, and the primary lesion was regarded as resectable. She underwent right hemicolectomy with lymphadenectomy as conversion therapy. The specimen could be removed without duodenectomy. Histopathologically, the tumor was 35 × 34 mm, tub2, ly0, v2, ypT3, ypN0, classified as stage IIA according to the Tumor Node Metastasis Classification of the International Union Against Cancer, version 7.0 [5]. The patient maintained normal carbohydrate antigen 19-9 (CA19-9) levels (<37 U/mL) and slightly high carcinoembryonic antigen (CEA) levels at 10 ng/mL (normal, <5.0 ng/ml) after the colectomy. However, two and half years after the colectomy, the serum CA19-9 level and CEA level increased to 635 U/mL and 22 ng/mL, respectively. CT revealed a mass measuring 20 × 20 mm in the small intestine (Fig. 1a). ^18^F-fluorodeoxyglucose-positron emission tomography/computed tomography (^18^FDG-PET/CT) revealed an abnormal uptake of ^18^FDG in that region, with maximum standardized uptake values of 5.7 (Fig. 1b). Double-balloon enteroscopy revealed an approximately 2-cm submucosal tumor in the jejunum at 100 cm from the incisor teeth. An irregular-shaped depression with a reddish color change was noted at the oral side of the lesion (Fig. 2). Histopathology of biopsy specimens from that lesion showed ectopic pancreas without malignancy. Therefore, peritoneal metastasis from colon cancer concomitant with ectopic pancreas or adenocarcinoma arising from ectopic pancreas was considered as a differential diagnosis. The patient underwent laparoscopic jejunectomy (resection of the jejunum, 20 cm) with regional lymph node dissection. Peritoneal metastasis was not observed. The patient’s postoperative course was uneventful. Gross examination revealed a rubbery mass measuring 28 × 20 mm, which had invaded the serosal surface of the jejunum. The cut surface of the tumor had a yellowish-white mass in the jejunal wall.

**Fig. 3:**
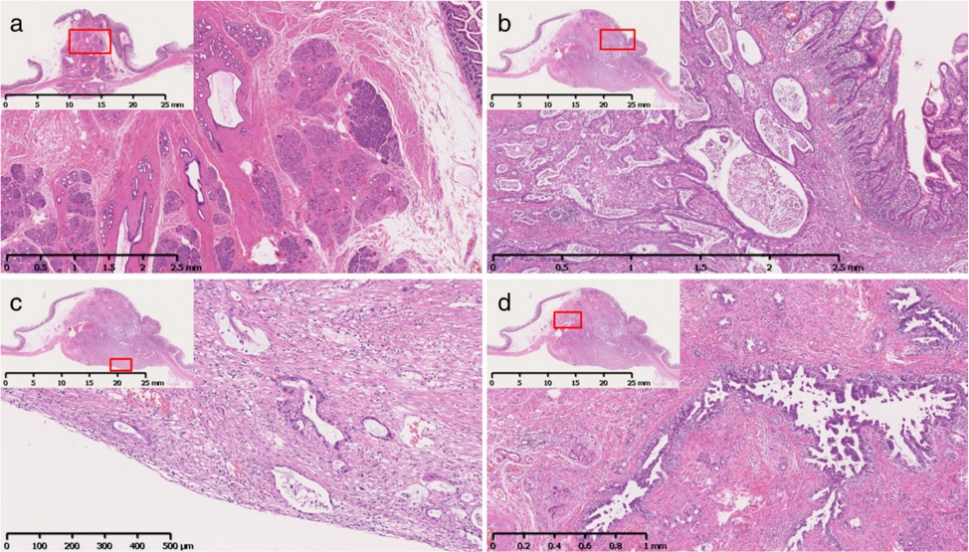
Histological findings of submucosal tumor. Pancreatic tissue consisting of acini and ducts was detected below the mucosal layer (**a**). Moderately differentiated adenocarcinomas proliferated diffusely in an area ranging from the submucosal layer (**b**) to the serosa (**c**) and were also found in the epithelium of the ducts (**d**)

**Fig. 4:**
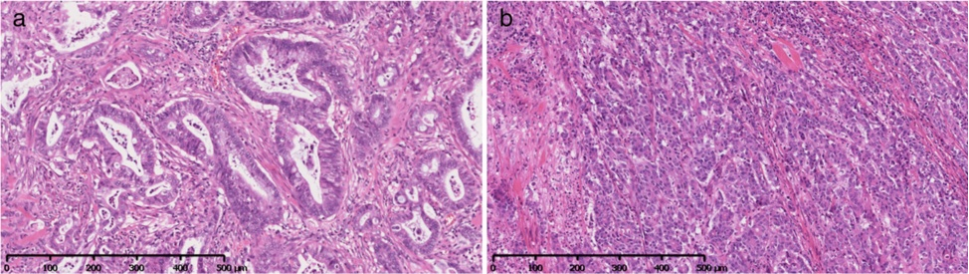
Microscopic view of resected submucosal tumor in the jejunum (**a**) was completely different from that of previously resected colon cancer (**b**)

The findings of histopathological examination were as follows: ectopic pancreatic tissue was detected below the mucosal layer, and consisted of acini and ducts, without Langerhans cells (Fig. 3a); moderately differentiated adenocarcinomas proliferated diffusely in an area ranging from the submucosal layer to the serosa (Fig. 3b, c); and they were also found in the epithelium of the ducts (Fig. 3d). This type of adenocarcinoma (Fig. 4a) was completely different from the previously resected colon cancer (Fig. 4b). Immunohistochemistry analysis was positive for cytokeratin 7 (CK7), mucin 1 (MUC1), and MUC5AC and negative for CK20, caudal-related homeobox gene 2 (CDX-2), and MUC6. These findings resulted in a diagnosis of adenocarcinoma arising from ectopic pancreas. A single lymph node located close to the tumor was found to be metastatic.

**Fig. 5:**
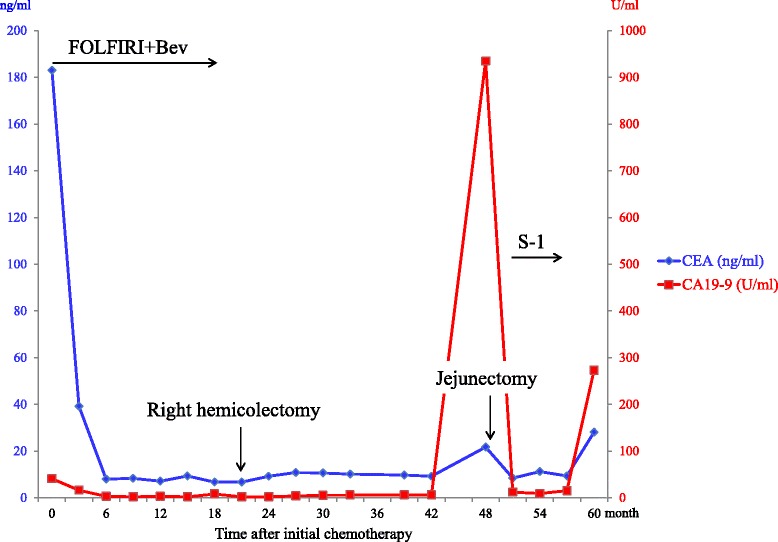
Chronological changes of the serum levels of CA19-9 and CEA. *FOLFIRI+Bev* folinic acid, 5-fluorouracil, irinotecan, and bevacizumab; *S-1* an oral fluoropyrimidine derivative

One month after surgery, the CA19-9 level decreased to normal, and an oral fluoropyrimidine derivative (S-1) started to be administrated as adjuvant chemotherapy, according to the protocol for treatment of resected pancreatic cancer [6]. However, the patient discontinued the chemotherapy because of adverse effects after receiving 2 cycles. Nine months after surgery, the serum CA19-9 level and CEA level increased to 273 U/mL and 28 ng/mL, respectively, and CT revealed multiple nodules in the abdomen with abnormal uptakes of ^18^FDG. Biopsy of these lesions was recommended to the patient; however, she rejected it. Peritoneal metastasis from ectopic pancreatic adenocarcinoma was suspected rather than peritoneal metastasis from colon cancer because the serum CA19-9 level was almost normal before treatment of colon cancer was begun (Fig. 5). Therefore, gemcitabine and nab paclitaxel were administrated for recurrent pancreatic cancer. After receiving 3 cycles, she maintained stable disease.

### Discussion

Ectopic pancreas is the presence of pancreatic tissue located outside the normal pancreas. It is observed in 0.5 % of upper abdominal laparotomies and 0.6 to 13.7 % of autopsy studies [1, 5]. It is preferentially found in the upper gastrointestinal tract (stomach 26 % and duodenum 28 %), followed by the small bowel (18 %), Meckel’s diverticulum (6 %), the colon, spleen, and gallbladder [7, 8]. The Heinrich classification is widely used for pathological classification of ectopic pancreas [9]. Heinrich type I contains ducts, acini, and islets. Heinrich type II is composed of ducts and acini, lacking islets. Heinrich type III consists of ducts and smooth muscle tissue only, lacking acini and islets. Our case lacked islets, so we classified the ectopic pancreas as Heinrich type II.

The incidence of malignant transformation of ectopic pancreas has been reported to range from 0.7 to 1.8 % [7, 8]. Moreover, ectopic pancreatic carcinoma is usually located in the stomach or the duodenum; ectopic pancreas in the jejunum is extremely rare [3, 8]. Criteria have been proposed for the diagnosis of carcinoma from an ectopic pancreas as follows: (1) the tumor must be located within or near the ectopic pancreas tissue, (2) transition between pancreatic tissue and carcinoma must be observed, and (3) the non-neoplastic pancreatic tissue must comprise fully developed acini and ducts [7]. Our case satisfied these three criteria. In immunohistochemical staining, CK7, MUC1, and MUC5AC were positive, and CK20 and CDX-2 were negative, which is in good agreement with the features of ordinary pancreatic adenocarcinoma [8–10]. The histology of ectopic pancreatic adenocarcinoma was completely different from the histology of colon cancer. Thus, these pathological findings were consistent with a diagnosis of adenocarcinoma arising from jejunal ectopic pancreas, not with a diagnosis of peritoneal metastasis from colon cancer.

In our case, the tumor markers, measured for surveillance of recurrence after resection of advanced colon cancer, were elevated. CT revealed a mass with abnormal uptake of ^18^FDG in that region in ^18^FDG-PET. First, peritoneal metastasis from colon cancer or primary small intestinal cancer was suspected. However, double-balloon enteroscopy revealed a submucosal tumor in the jejunum, and histopathology of the biopsy specimens showed ectopic pancreas without malignancy. On the basis of the findings of elevated tumor marker and abnormal uptake of ^18^FDG, peritoneal metastasis from colon cancer with ectopic pancreas or adenocarcinoma arising from ectopic pancreas was considered as a differential diagnosis; therefore, resection of that lesion was required.

We performed a PubMed search of the English literature from 1966 to 2014 using the following keywords: ectopic, aberrant, heterotopic, pancreas, jejunum, small intestine, adenocarcinoma, and cancer. We also retrieved cases from the relevant reference lists. We found six previous reports of adenocarcinoma arising from jejunal ectopic pancreas (Table 1) [3, 11–15]. Six cases other than our case had abdominal symptoms. There were no cases diagnosed as adenocarcinoma arising from ectopic pancreas preoperatively. The serum CA19-9 level was predominantly elevated compared to the elevation of the serum CEA level, which was consistent with ordinary pancreatic cancer [16]. Lymph node metastasis was observed in two cases. There has been no evidence of the efficacy of chemotherapy for adenocarcinoma arising from gastrointestinal ectopic pancreas. Fukino et al. reported a case in which gemcitabine was effective for recurrent adenocarcinoma arising from duodenal ectopic pancreas [4]. The prognosis of adenocarcinoma arising from ectopic pancreas is also unclear. A few patients were reported to be alive without recurrence more than 5 years after resection of adenocarcinoma arising from ectopic gastric or duodenal pancreas, and reports have also indicated a more favorable prognosis than that for ordinary pancreatic cancer because of its earlier presentation [17–20]. However, in our literature review, four patients with adenocarcinoma arising from jejunal ectopic pancreas experienced distant metastasis, and survival time ranged from 5 to 9 months. As for adenocarcinoma arising from jejunal ectopic pancreas, the prognosis seems to be worse than that for adenocarcinoma arising from other gastrointestinal ectopic pancreases. This might result from differences in lymph node dissection. For adenocarcinoma arising from gastric or duodenal ectopic pancreas, lymph node dissection is performed according to the presence of gastric cancer or duodenal cancer. However, as for the small intestine, the efficacy and appropriate extent of lymph node dissection are unknown. More cases need to be investigated to provide some answers.

**Table 1:** Reported cases of adenocarcinoma arising from jejunal ectopic pancreas

Case	Year	Age (years)	Sex	Opportunity to diagnose	CA19-9 (U/mL)	CEA (ng/mL)	Size (mm)	Heinrich type	Lymph node metastasis	Adjuvant chemotherapy	Prognosis	Reference
1	1988	85	F	Bowel obstruction	ND	ND	15	ND	ND	ND	ND	11
2	1999	63	M	Bowel obstruction	6100	17	40 × 20	II	ND	ND	ND (peritoneal carcinomatosis and hepatic metastasis at surgery)	12
3	1999	61	M	Bowel obstruction	ND	ND	15	I	ND	ND	ND	13
4	2008	64	F	Bowel obstruction	ND	ND	20	ND	ND	Gemcitabine	Death (5 months) (peritoneal carcinomatosis)	14
5	2010	76	F	Bowel obstruction	76	Normal	20 × 15	III	Positive	ND	Death (5 months) (hepatic metastasis)	3
6	2012	74	M	Diffuse peritonitis	88	Normal	30	II	ND	ND	ND	15
Present case	2015	69	F	Elevation of tumor marker	635	22	20 × 20	II	Positive	S-1	Not death (9 months) (peritoneal carcinomatosis)	–

Normal levels of CA19-9 and CEA are below 37 U/mL and 5 ng/mL, respectively

*M* male, *F* female, *ND* not described, *S-1* an oral fluoropyrimidine derivative

## Conclusions

In summary, we report a rare case of adenocarcinoma arising from jejunal ectopic pancreas after resection of advanced colon cancer. Even if histopathology of the biopsy specimen shows ectopic pancreas without malignancy, adenocarcinoma arising from ectopic pancreas should be considered when the tumor markers are elevated or the lesion has an abnormal uptake of ^18^FDG.

## Consent

Written informed consent was obtained from the patient for publication of this case report and any accompanying images. A copy of the written consent is available for review by the editor in chief of this journal.

## Abbreviations

^18^FDG-PET: ^18^F-fluorodeoxyglucose-positron emission tomography
CA19-9: carbohydrate antigen 19-9
CDX: caudal-related homeobox gene
CEA: carcinoembryonic antigen
CK: cytokeratin
CT: computed tomography
FOLFIRI+Bev: folinic acid, 5-fluorouracil, irinotecan, and bevacizumab
MUC: mucin
